# Propolis Counteracts Some Threats to Honey Bee Health

**DOI:** 10.3390/insects8020046

**Published:** 2017-04-29

**Authors:** Michael Simone-Finstrom, Renata S. Borba, Michael Wilson, Marla Spivak

**Affiliations:** 1 USDA-ARS Honey Bee Breeding, Genetics, and Physiology Laboratory, Baton Rouge, LA 70820, USA; 2 Centre for High-Throughput Biology, University of British Columbia, Vancouver, V6T 1Z4, Canada; renata.borba@canada.ca; 3 Beaverlodge Research Farm, Agriculture and Agri-Food Canada, Beaverlodge, AB T0H 0C0, Canada; 4 Center for Drug Design, University of Minnesota, Minneapolis, MN 55018, USA; wils0888@umn.edu; 5 Department of Entomology, University of Minnesota, St. Paul, MN 55108, USA; spiva001@umn.edu

**Keywords:** *Apis mellifera*, social immunity, plant resin, plant insect interactions

## Abstract

Honey bees (*Apis mellifera*) are constantly dealing with threats from pathogens, pests, pesticides and poor nutrition. It is critically important to understand how honey bees’ natural immune responses (individual immunity) and collective behavioral defenses (social immunity) can improve bee health and productivity. One form of social immunity in honey bee colonies is the collection of antimicrobial plant resins and their use in the nest architecture as propolis. We review research on the constitutive benefits of propolis on the honey bee immune system, and its known therapeutic, colony-level effects against the pathogens *Paenibacillus larvae* and *Ascosphaera apis*. We also review the limited research on the effects of propolis against other pathogens, parasites and pests (*Nosema*, viruses, *Varroa destructor*, and hive beetles) and how propolis may enhance bee products such as royal jelly and honey. Although propolis may be a source of pesticide contamination, it also has the potential to be a detoxifying agent or primer of detoxification pathways, as well as increasing bee longevity via antioxidant-related pathways. Throughout this paper, we discuss opportunities for future research goals and present ways in which the beekeeping community can promote propolis use in standard colonies, as one way to improve and maintain colony health and resiliency.

## 1. Introduction

Honey bee (*Apis mellifera*) populations in North America and Europe are currently experiencing high annual losses throughout the year due to various, often interacting factors including pathogens, parasites, pesticides, poor nutrition and management [1,2,3,4]. It is critically important to understand the impact of individual stressors and the interactions among stressors in order to develop solutions to increase colony health and survival. It is equally important to understand how honey bees’ natural immune responses (individual immunity) and collective behavioural defenses (social immunity) can improve and maintain bee health and counteract stressors without human intervention. One form of social immunity in honey bee colonies is the formation of a propolis envelope within the nest that acts as an important antimicrobial layer. While a propolis envelope cannot mitigate all colony stressors, we review research to date on its known benefit to individual immunity and effect on reducing colony pathogen loads. We also suggest research avenues that could reveal additional ways propolis may improve colony health and resiliency.

### 1.1. The Role of Propolis in the Hive

Propolis is the apicultural term for the plant resins that honey bees collect, bring back into the hive, and then deposit throughout the nest [5]. Resin-handling bees within the nest mix varying amounts of wax, which they produce themselves, with the resins during deposition [5,6]. The primary chemical components of propolis are derived from the plant-produced resins, though there is some evidence that honey bee glandular secretions (i.e., β-glucosidase) may also be added, potentially as an artifact of handling [7]. Various species across Hymenoptera (e.g., ants [8], stingless bees [9], and other *Apis* species [10,11]) collect and use resins for various purposes, including nest construction, and as a defense against predators, microbes and pathogens.

**Figure 1:**
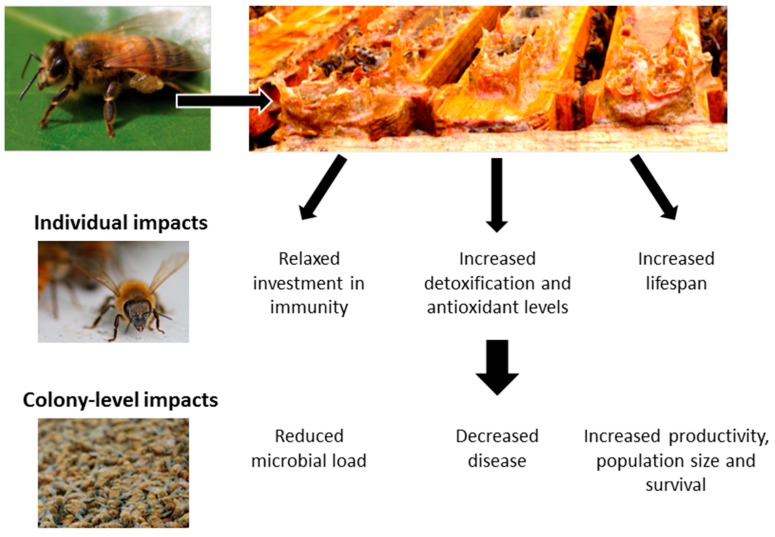
Overview of the impacts of honey bee-harvested plant resins on individual and colony health (photos by Michael Simone-Finstrom).

Honey bee colonies typically nest in tree hollows. Once a swarm (thousands of non-reproductive workers and one queen) finds a suitable cavity, they line the interior walls and often the nest entrance with a layer of propolis 0.3–0.5mm thick [10]. This propolis envelope surrounds the colony and likely serves many non-mutually exclusive purposes, including waterproofing and preventing fungal decay of the hive walls [5,12], reducing cracks and hive entrances, helping promote stable temperature and humidity through condensation [13], and reducing hive microbial loads [5,14]. In addition, there have now been several, mainly recent, studies examining the indirect and direct effects that propolis has on bees’ immune systems, pathogens and parasites [5,14,15,16,17,18,19,20,21,22] (Figure 1).

### 1.2. Propolis as Social Immunity

Social immunity describes cooperative behavioral defenses among members of a social group that result in the avoidance, control or elimination of parasitic infections [23]. A social immune system supplements and enhances the individual immune system, benefitting the health of a colony by decreasing the risk of microbe exposure and disease transmission among group members [24].

The nests of densely populated honey bee and other social insect colonies provide a favourable habitat for a wide range of parasites and pathogens [25] that have evolved to overwhelm or suppress their hosts’ immune defenses. Honey bees were perhaps not subject to the multitude of pathogens and other stressors that confront them today within the context of modern beekeeping practices (e.g., transportation of hives, density of hives in close proximity, and exchange of comb across beekeeping operations [3]), but, as cavity nesters within trees, they likely encountered saprophytic, symbiotic and parasitic microbes and fungi. Some of these endemic microbes can induce an immune response in individual bees [26,27]. The evolution of resin use by honey bees could have been to combat fungal growth and potential fungal pathogens within the nest. Although both bacteria and fungi are common bee immune stressors, the honey bee immune system appears to be more attuned against bacterial pathogens [28]. One hypothesis is that social immune behaviors, such as the collection of plant resins and their deposition in the nest as an antimicrobial propolis envelope, evolved to compensate for deficiencies in innate or physiological immunity. Supporting this notion, there is some evidence that propolis may reduce impacts of mycotoxins produced by fungi [29]. In this case, feeding propolis extracts, rich in flavonoids and phenolic compounds, to adult honey bees reduced adverse effects of exposure to toxins produced by *Aspergillus*, a common hive fungus.

The immune system is the most costly physiological system in insects [30]. An elevated immune response can lead to reduced colony productivity in honey bees [31] and decreased individual survival in bumble bees [32]. Thus, it is not surprising that honey bees have evolved behavioral mechanisms of social immunity to reduce activation of the individual immune system against these microbes to ultimately benefit colony health [33,34,35].

## 2. Constitutive Effects of Propolis on the Honey Bee Immune System

**Figure 2:**
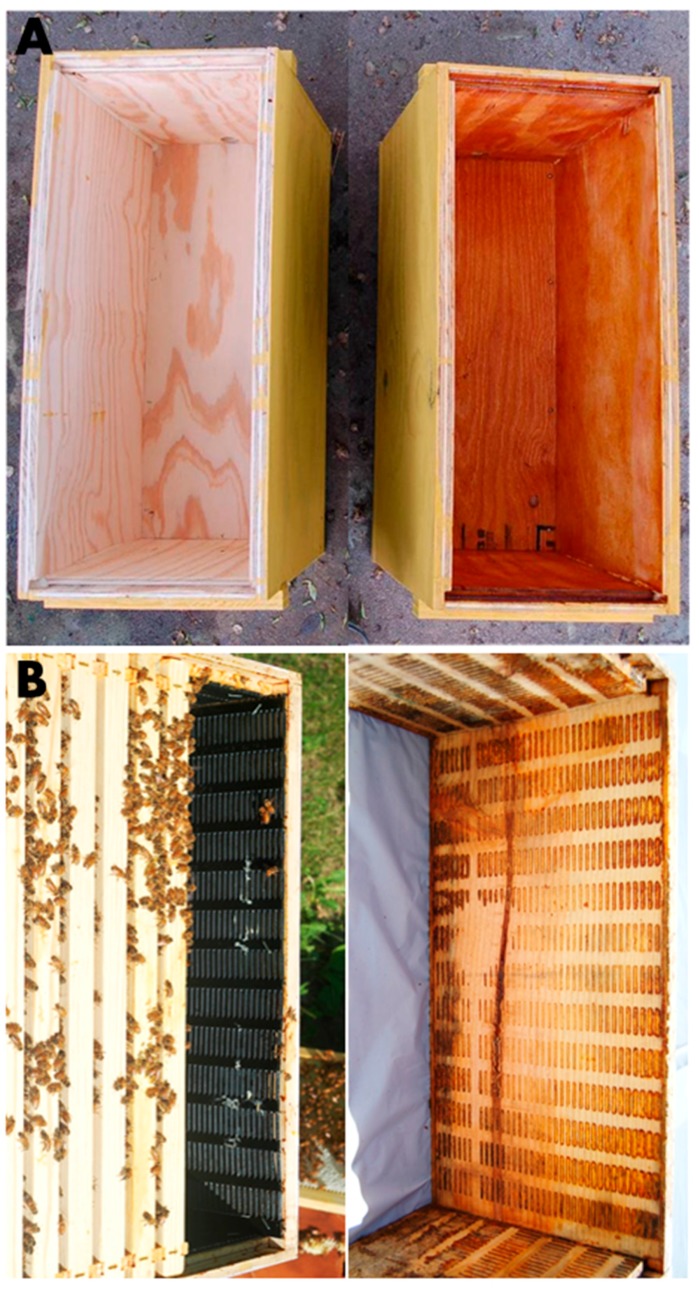
Examples of propolis envelopes in managed hives. (**A**) a propolis extract was used to “varnish” the interior hive body walls. The left was treated with 70% ethanol, the solvent for the extract, while the right was treated with poplar-derived propolis extract (photos by Michael Simone-Finstrom). (**B**) commercially purchased “propolis traps” were cut and stapled to the interior walls (**left**). Once the bees deposit sufficient propolis in the trap, the trap can be removed leaving the propolis attached to the wall (**right**; photos by Renata S. Borba).

Our experiments on the effects of resin on honey bee health followed findings of other researchers on the use of resin by social wood ants (*Formica paralugubris*) in Switzerland. This ant species constitutively collects spruce tree resins and places globules of resin near the brood, which results in reduced growth of microorganisms in the nest mound [8,36], lower immune system activity of adult worker ants [37], and increased survival during pathogen challenge [38]. Based on these findings, we hypothesized that, for a honey bee colony, the antimicrobial properties of propolis would reduce the general microbial load within the nest cavity, thereby reducing the production of antimicrobial peptides by the innate immune system of individual bees. The first study to test if propolis exposure influenced honey bee immune function confirmed our prediction [14]. Bees were hived in small man-made bee boxes, enriched with an experimentally applied propolis envelope (ethanol extract of propolis painted inside the hive box; Figure 2A). Extracts were made from propolis previously collected from colonies at the University of Minnesota (resins collected mostly from plants in the genus *Populus* [17]), or from Brazilian green propolis (from the tropical shrub *Baccharis dracunculifolia* [39]). General bacterial loads (16S rRNA expression) and transcription levels of the gene encoding for the antimicrobial peptide, *hymenoptaecin*, and a gene involved in cellular immunity, *AmEater*, were found in significantly reduced quantities in seven-day-old bees from colonies with the propolis-extract envelope compared to bees in colonies with no propolis envelope. In summary, bees in colonies with the propolis-extract envelope had an overall reduction in general bacterial loads, and a corresponding lower investment in immune gene expression compared to bees in boxes without the propolis envelope [14].

A second study [20] also confirmed the prediction that bees in colonies with a propolis envelope had reduced investment in individual bee immune function, but, in contrast to Simone et al. [14], no reduction in overall bacterial load was observed. The methods of the second study differed: rather than painting a propolis-extract envelope within the bee boxes, the colonies were allowed to deposit their own propolis envelope from resins collected within a foraging range of the University of Minnesota. Twenty-four colonies were hived from “packages” (10,000 bees and a mated queen) in new Langstroth bee boxes. Twelve of the colonies were hived in boxes fitted with commercial propolis traps (plastic grids) along all inner walls of the brood boxes, which stimulated the bees to form a propolis envelope (Figure 2B). The effect of the propolis envelope on colony strength and health, and on individual bee immune function was measured over a full year. As a validation of our methods, the entire experiment was repeated a second year using 24 new colonies. The expression of immune-related genes, particularly *hymenoptaecin* and *abaecin*, of individual seven-day-old bees was significantly lower over the entire foraging season in colonies with a propolis envelope in both trials, indicating the long-term effect of the propolis on baseline expression of immune-related genes [20]. By the following spring, there were no significant differences in gene expression levels for most immune genes between bees from the two treatment groups. The effect on the immune system was diminished [20], likely due to the decrease in biological activity of the propolis during the winter months when bees were not foraging or seasonal variation in availability of resin sources in early spring, which can vary in active compounds [20,40,41]. Presumably, when the growing season commenced, bees would add new propolis on top of the old and restore the antimicrobial properties of the envelope. Bees in colonies with a propolis envelope did have significantly higher levels of the blood storage protein vitellogenin (Vg) compared to control colonies in spring of both years. Higher levels of Vg are an indicator of well-nourished bees [42,43,44]. A decrease in energetic costs associated with the maintenance of an efficient immune system during the foraging season might help bees maintain higher storage protein levels (e.g., vitellogenin) required for overwintering success [45] and allocate energy to perform vital tasks the next spring (e.g., foraging, rearing brood).

The results of these two experiments demonstrated that costly immune gene expression in bees was lowered by the presence of the propolis envelope within the nest, but clear effects of propolis on colony fitness were not observed. We did not measure the effects of propolis envelope on individual bee fitness, which, in retrospect, may have been more revealing. Nicodemo et al. [18] found positive effects of propolis on individual bee fitness by measuring brood viability and adult bee longevity of Africanized honey bee colonies, an *A. mellifera* subspecies notable for depositing high amounts of propolis in some naturally selected colonies and those bred for increased propolis use due to industry export demands [18,46,47,48]. Colonies selected for high propolis collection for one generation produced 34 times more propolis compared to colonies selected for low propolis collection, consistent with the high heritability estimates for propolis collection ranging from 0.66 [49] to 0.87 [46]. Brood survival rates (survival from egg to adult stage) and individual adult bee longevity were both significantly higher in the high-propolis collecting colonies. The effects of propolis on colony-level measures have been less clear [46,47,48,49]. High propolis collecting colonies had more pollen and honey stores within the nest in some experiments [47,48,49] (but see [46]); it is unclear, however, if high propolis collection was correlated with foraging rates in general in these experiments. Bees within the high propolis collecting colonies also exhibited increased hygienic behaviour [46,48,49], assayed by the rate of removal of pin-killed brood (a proxy for removal of diseased, parasitized and dead brood), although there were no differences in levels of *Varroa* mites between sets of colonies [49].

Future studies might continue the work of Borba et al. [20] to examine the effect of the propolis envelope on better metrics of colony-level fitness such as queen longevity or colony reproduction (swarming) and survivorship, particularly for European honey bees. Furthermore, the potential benefits of propolis enrichment for colonies in a commercial, migratory or honey production operation need to be explored more fully. For the effects of propolis on the fitness of individual bees, experiments could investigate whether the reduced investment in immune function by individual bees after exposure to propolis corresponds with increased bee weight at emergence and longevity.

## 3. Does Propolis Suppress Immune Function?

**Figure 3:**
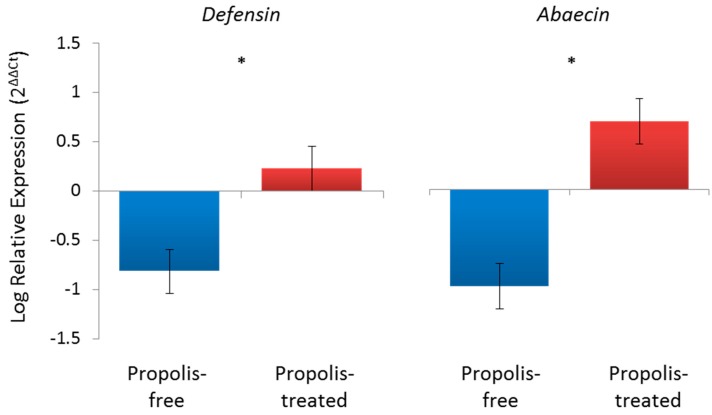
Propolis exposure may boost individual immunity when challenged. The log relative expression was calculated using the ∆∆Ct method [54] with expression for LPS-fed bees is shown relative to expression of unchallenged control bees for the propolis-free and propolis-treated cages. Expression of the antimicrobial peptides is relative to the reference *β-actin*. The propolis-treated cages resulted in a significant increase in immune gene expression in LPS-fed, immune-challenged bees for both *defensin* (*F*_1,31_ = 12.46, *p* = 0.001) and *abaecin* (*F*_1,31_ = 9.06, *p* = 0.005), as indicated by the “*” between treatment groups. *n* = 15 bees per treatment group.

**Figure 4:**
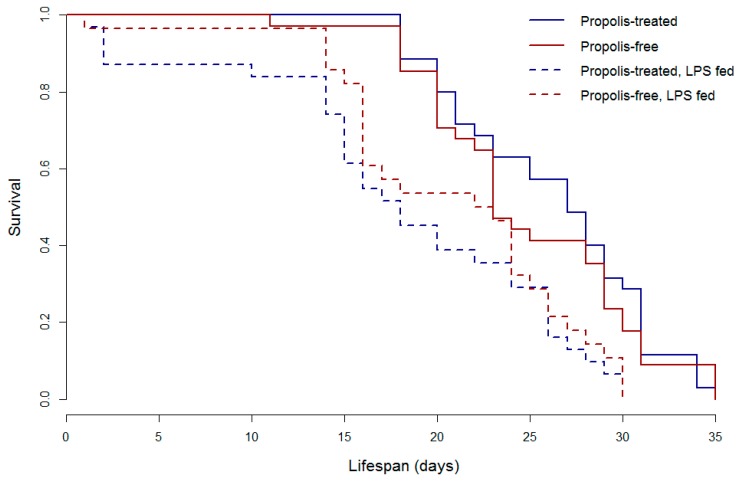
Immune challenge reduces honey bee lifespan in caged bees (Wald Χ^2^ = 14.18, *p* = 0.002). In the incubator setting, which is likely more stressful than a hive, there was no effect of the propolis enriched environment on lifespan (*p* = 0.83). *n* = 35 bees per treatment group.

The reduction in immune function observed in bees surrounded by a propolis envelope [14,20] does not imply the bees’ immune system was suppressed or inhibited. A recent study using honey bees maintained in “bee cups” coated with propolis extracts collected from North Dakota or left propolis-poor by coating with an equal amount of 70% Ethanol (the solvent of the extracts), confirmed that propolis does not cause immune suppression [50]. Individual, newly emerged bees were fed either 2 µL of sucrose solution (unchallenged controls) or 2 µL of sucrose containing 40 mg of lipopolysaccharides (LPS) from *Escherichia coli* (challenged). LPS is the major outer surface membrane component of Gram-negative bacteria and a known immune-stimulant for honey bees (and other insects [51,52]), and, as such, acts to activate the immune system without creating an active infection. Analysis of immune gene expression of individual bees from the cages showed bees exposed to propolis from North Dakota had an enhanced immune response in challenged individuals relative to unchallenged controls for the genes encoding the antimicrobial peptides *defensin1* and *abaecin* (Figure 3; [50]). Unchallenged control or challenged LPS-fed bees were also monitored for survival. Immune activation via LPS significantly reduced lifespan of all bees, but there was no effect of propolis exposure (Figure 4; [50]). While, in this controlled cage study, a general reduction in immune gene expression was not observed as in colony-level field exposures [14,20], this could be due to the sterile environment of the incubator cages. Earlier work suggested that the reduction in immune gene expression could be, at least in part, due to the reduction of hive microbes [14] (but, see [20]), which would not be present in the incubator cages. In summary, the challenged bees in a propolis-enriched environment had an increased immune response compared to challenged bees in the cage environment with no propolis, indicating that the presence of propolis does not appear to directly suppress the immune system. Recent evidence suggests that at least some components of propolis may be able to influence lifespan in the cage setting, as bees fed with *p*-coumaric acid and quercetin, common phytochemicals in hive products like propolis, had longer lifespans compared to control bees under specific conditions. Though exposure to propolis volatiles in the cage study did not affect lifespan (Figure 4), possibly due to smaller samples sizes than those in the recent cage study with oral treatments [53], it remains to be verified if propolis exposure or hive supplementation in general increases lifespan of individual bees under more realistic conditions, as observed by Nicodemo et. al. [18] in Africanized honey bees bred for increased propolis production.

Bees’ exposure to propolis may also “prime” the immune system in the way that it appears to prime detoxification pathways, at least for experiments involving oral doses of propolis extracts or chemical components [29,55,56]. In another field-based experiment, colonies with and without a natural propolis envelope were either challenged or not with spores of a honey bee bacterial pathogen, *Paenibacillus larvae* (see section below for details, [19]). In the presence of clinical signs of the disease, adult bees showed significantly higher expression of two antimicrobial peptides, *hymenoptaecin* and *apidaecin*, compared to bees in colonies without a propolis envelope. This further provides support that the immune system is not supressed when bees are exposed to propolis, but simply can allow for a reduced investment in immune expression when unchallenged [14,20]. In fact, bees in an environment with propolis invested more in the production of antimicrobial peptides when the colony was exposed to pathogenic microbes compared to same-aged bees in colonies without propolis [19], similar to what was found in the cage study (Figure 3). Individual investment in inducible immune defences (e.g., antimicrobial peptide production) therefore may be at least somewhat context dependent on the colony investment in constitutive defenses (i.e., propolis deposition; [34]), given that reliance on both types of broad immune defenses may also be regulated by type of pathogen exposure [57]. Thus, the propolis envelope can be viewed as an external and vital component of the bees’ immune defense and the colony’s social immunity.

## 4. The Effect of Propolis against Pathogens

The majority of propolis-related research has focused on identifying chemical components, and understanding the bioactivity of whole extracts, fractions and specific compounds. Much of this is in relation to identifying novel compounds that could be used for human health applications [58,59,60,61,62]. Several reviews have examined this subject over the years, consistently updating the numbers of new chemical compounds being discovered in propolis samples collected across the globe and often including measures of biological activity. Over 300 compounds with variable biological activities have been identified in propolis samples [63] (see Box 1).

Box 1. **Highlight of recent work to identify the major bioactive components of propolis**

Propolis is a rich and diverse source of biologically active compounds due to the diversity of resin-producing plants throughout the world [64]. Propolis has been widely investigated for compounds useful to human health and is regularly screened for cytotoxicity, antibacterial, and antifungal activity. In addition, propolis has been studied for its effects on the immune system and anti-protozoal activity. Several flavonoids and their esters isolated from Sonoran propolis induce apoptosis in a β-cell lymphoma cell line [65], while the polyisoprenylated benzophenone nemorosone from *Clusia*-derived Cuban propolis is cytotoxic against four different cancer cell lines [66]. Prenylated cinnamic acids isolated from Brazilian propolis [67] and terpenes isolated from both Brazilian propolis and Cretan propolis [68] have reported antimicrobial activity against bacterial and/or fungal pathogens. These compounds from Brazilian and Cretan propolis originated from *Baccharis dracunculifolia* and likely *Cupressaceae* conifers, respectively [69]. Caffeic acid phenethyl ester (CAPE) from poplar-derived propolis has been a target of much interest, as it appears to be a major factor modulating anti-inflammatory and immunomodulatory properties of some propolis varieties [61]. Lastly, diterpenes from Libyan propolis were found to have antiprotozoal activity against *Trypanosoma brucei* and *Leishmania donovani* [70].

Recent studies have also focused on propolis compounds important for bee health, with an emphasis on brood diseases. Several known compounds with activity against *P. larvae* have been isolated from Bulgarian propolis including pinocembrin, pinobanksin-3-acetate, and a mixture of caffeic acid esters [71]. However, these compounds may not be responsible for the majority of anti-*P. larvae* activity observed in U.S. propolis samples [21]. Using bioassay-guided fractionation, Wilson et al. [72] isolated pinobanksin-3-octanoate and five other 3-acyl-dihydroflavonols that showed inhibitory activity against both *P. larvae* and *A. apis.* These 3-acyl-dihydroflavonols originated from *Populus fremontii* resin, but varying amounts were also found in other North American *Populus* resins. The amount of pinobanksin-3-octanoate and pinobanksin-3-hexanoate in regional samples of U.S. propolis was strongly correlated with anti-*P. larvae* activity, suggesting that these compounds could be markers of anti-*P. larvae* activity. Voigt and Rademacher [73] found that the common propolis phenolics cinnamic acid and pinocembrin had a fungistatic effect on *A. apis*. We do not understand how propolis compounds interact with bee pathogens in the hive, so this question should be a priority in future studies.

Propolis is most well-known for its antimicrobial activity against a number of bacterial, fungal and viral pathogens, particularly human pathogens [74,75,76,77]. A honey bee colony collects resin from multiple plant sources, though there is likely source fidelity for individual bees [5,17]. The antimicrobial activity of the propolis depends on the antimicrobial properties of its source plant resins [21,72], and the fact that colonies mix sources of resins creates an extremely complex mixture against which susceptible parasites and pathogens would have difficulty developing resistance. A previous study investigating the biological activity of single and multiple-resin source against stingless bee parasites and pathogens showed that, although some single-resin sources may be more effective against a single parasite or pathogen, mixed sources are considerably more effective overall due to increased variety of chemical compounds [78].

The therapeutic value of propolis for honey bees has been understudied. Most studies have concentrated on the high in vitro activity of propolis from various regions of the world against two pathogens: *Paenibacillus larvae*, the causative agent of American foulbrood (AFB) [15,19,21,79,80,81]; and *Ascosphaera apis*, the fungal agent of chalkbrood disease [16,21,73]. Early studies on the therapeutic effects of propolis on individual bees and colonies yielded positive results when propolis was fed to the bees in sugar solution (e.g., [80,81]). In these studies, clinical signs of AFB in field colonies, and number of *P. larvae* spores in honey stores were reduced when bees were fed propolis in sugar syrup. Feeding bees propolis would be similar to administering oral antibiotics, but as the antimicrobial properties of propolis vary widely across plant sources and regions, oral application risks under- or over-dosing the bees and potentially harming the beneficial microbiota in bees’ guts [82]. To our knowledge, honey bees do not naturally consume propolis. Therefore, the mode of action of a therapeutic effect of propolis on colony pathogens is probably via volatile compounds [83] or direct contact [84] either on the hive walls, a barrier at the nest entrance, or along the rims of comb cells.

Our in vivo studies at the colony level first examined the effectiveness of the propolis envelope within the nest to reduce chalkbrood infection [16]. Chalkbrood is one of the major fungal diseases affecting honey bees, which only infects the larval stage [85]. Adult bees are carriers of the fungal spores and feed young larvae contaminated brood food, presumably by adding nectar from the bee crop contaminated with spores into brood food. Alternatively, the food fed to young larvae may become contaminated with fungal spores left in the wax comb from a previous infection in the colony. As the larva develops, the spores germinate in the gut and eventually penetrate into the hemocoel, overwhelming the bee with mycelia and turning it into a “chalkbrood mummy.” In one field test of potential effects of propolis against chalkbrood, colonies were either enriched with an experimentally applied propolis envelope (ethanol extract of Minnesota-derived propolis painted inside the hive box) or not propolis-enriched (as in Figure 2A). Colonies were then challenged by feeding them pollen patties containing *A. apis* spores, prepared by homogenizing chalkbrood mummies in the pollen [86]. After three weeks, the colonies with a propolis envelope had significantly fewer chalkbrood mummies in the combs (14.7 + 7.5 mummies; *n* = 6 colonies), compared to colonies without the propolis envelope (108.2 + 40.0 mummies, *n* = 6 colonies) [16]. One known mechanism of resistance to chalkbrood disease is through hygienic behaviour, whereby the adult bees detect and quickly remove chalkbrood-infected brood from the nest before the pathogen becomes infectious [86]. In this experiment, none of the colonies were considered hygienic, as determined by freeze-killed brood assay (reviewed in [33]); thus, the difference in disease levels between the two sets of colonies was not due to differences in hygienic behaviour. While in vitro work has shown that propolis extracts do directly inhibit growth of *A. apis* in liquid media [21], the mode of action for how the propolis extract painted on the inside of the bee box reduced fungal infection in the larvae, maintained mainly in the center of the nest, remains unknown.

In subsequent tests, the effectiveness of the propolis envelope to reduce severity of AFB, which is similar in etiology to chalkbrood disease, was examined [19]. Adult bees are only the carriers of *P. larvae* spores and are not susceptible to infection. As with chalkbrood, young larvae may become infected via contaminated larval food fed by adult bees, or the larval food may become contaminated with spores already in the wax comb. Bees resist AFB infection in a number of ways: in addition to hygienic behaviour of adult bees toward infected larvae [87,88], some larvae demonstrate genetic resistance against *P. larvae* [89,90], and young adult bees from some genetic lines secrete antimicrobial compounds into larval food, which protect the larvae from infection [91,92]. Our experiment consisted of challenging non-hygienic colonies in the field by spraying combs with sugar solution that contained spores of *P. larvae* (following [93,94]). Five challenged colonies were fitted with commercial propolis traps along all inner walls of the brood boxes, stimulating the bees to form a natural propolis envelope as in Borba et al. [20] (Figure 2B), and five other challenged colonies were not provided with propolis traps and did not construct a propolis envelope. We investigated the effects of the propolis envelope on the overall reduction of clinical signs of AFB, and on the antimicrobial activity of larval food fed to 1–2 day old larvae. The presence of the propolis envelope did not completely clear AFB infection; all colonies had clinical signs two months later at the end of the experiment, similar to early studies (e.g., [80]). However, the severity of AFB, in colonies with a propolis envelope was relatively mild (severity score just over 1, equating to 1–5 infected larvae per comb, following [87]) compared to the colonies without the propolis envelope (severity score just over 2, or 6–25 infected larvae per comb). Moreover, the ability of the larval food from challenged colonies with a propolis envelope to inhibit the growth of *P. larvae*, in vitro, was significantly higher compared to the activity of larval food from challenged colonies without a propolis envelope [19]. It is unclear if the increased antimicrobial activity of the larval food was due to the presence of antimicrobial peptides produced by adult bees and incorporated into larval food, or to the presence of compounds from the propolis in the food. Since antimicrobial peptides and other antimicrobials (e.g., glucose oxidase) are secreted by nurse bees into brood food, the same mechanism that allows adult bees to alter investment in innate immunity may allow nurse bees to invest more in these compounds as a social immune defense [95]. These studies emphasize the critical importance of the propolis envelope to honey bees’ health and demonstrate its role in larval defense against bacterial infections.

Field experiments have also provided evidence of self-medication in honey bees as the rate of resin foraging increased in colonies after challenge with the fungal pathogen, *A. apis*. Colonies have been shown to consistently increase the number of resin foragers after a colony-level infection with *A. apis* over several years of study [16,19]. This case of self-medication is particularly interesting because it occurs at the colony level and does not simply involve individuals ingesting compounds to self-medicate themselves [96]. Because only larvae are infected by *A. apis*, the increased resin collection is a great example of a social immune response increasing collection of antimicrobial products by adult bees to protect younger nestmates. Subsequent studies have determined that this self-medication behavior is pathogen-specific and does not occur for colony-level infections with the bacterial brood disease AFB [16,19]. This finding makes the response to chalkbrood all the more interesting and raises questions related to the mechanisms regulating the behavioural change and how colonies may differentially invest in social and physiological immune defences against fungal versus bacterial pathogens.

## 5. Effect of Propolis on Other Bee Pathogens, Parasites and Pests

Honey bee colonies are susceptible to various pathogens, parasites and pests [85,97]. The main health threats contributing to colony losses include the microsporidian gut parasite *Nosema* spp., the ectoparasitic mite *Varroa destructor*, and some of the 20 viruses known to infect bees [97]. Other secondary pests can kill weakened hives and destroy hive products, such as the greater wax moth, small hive beetle, and small rodents.

### 5.1 Nosema Ceranae

Nosema ceranae is a gut parasite of honey bees. This parasite was originally found in Asian honey bees, *Apis cerana*, and recently described in European honey bees, *A. mellifera* [98]. In the last 15 years, *N. ceranae* has spread throughout Europe and the U.S and largely replaced *N. apis* in these two regions [98,99]. To our knowledge, no studies have investigated the effects of propolis on *Nosema* spp. infection in honey bees (*A. mellifera*). However, two studies have investigated the effect of stingless bee (*Trigona apicalis*) propolis on *N. ceranae* infections in *A. cerana* [100] and *A. florea* [101]. While the methods used in these experiments are not ideal to fully answer the question because of the origin of propolis and the propolis extraction methods, they raise interesting questions that should be explored further. In both studies, individual bees were inoculated per os with sucrose solution containing spores of *N. ceranae*. After inoculation, bees were fed propolis extract in water. Similar results were found in both experiments: all challenged bees treated with propolis lived longer than untreated challenged bees (but not longer than unchallenged bees), and those fed with a high concentration of propolis (a 50% solution of propolis extract—60 g propolis/100 mL ethanol—in sugar syrup) had lower infection rate [100]. These studies provide a starting point to investigate the biological relevance of these findings within an *A. mellifera* colony and how propolis in the nest environment may also be impacting gut parasites. The results indicate that propolis may have toxicity to this gut pathogen, but also raises concerns about toxicity to beneficial microbiota in bees’ guts, and whether the natural mode of action is per os. This issue is of particular relevance since the bee microbiome consists of a large proportion of gram-positive bacteria [102], and propolis is generally more effective against Gram-positve bacteria verus Gram-negative bacteria [41].

### 5.2 Varroa Destructor

Of considerable interest is whether propolis has biological activity against *Varroa destructor*. A series of laboratory assays have shown that directly exposing mites held in Petri dishes to relatively low concentrations of ethanolic propolis extracts causes high mite mortality [22,84,103,104,105]. Ethanolic extracts from German propolis caused 100% mortality due to contact with 10% propolis extract [84]. Furthermore, mite exposure to extracts at concentrations as low as 0.5% caused narcotic effects leading to reduced heat production and metabolic rates [84,103]. Sublethal effects of low concentrations of propolis may debilitate a *Varroa* mite’s mobility, as well as make them less capable of dealing with environmental stressors (e.g., high temperatures [103]). Moreover, ethanolic extracts from Argentina also caused narcotic effects and significantly higher mite mortality (60.5% to 90%) compared to control treatments [105]. However, a recent study using raw propolis, not extracts, found no effect of propolis exposure on mite survival in the laboratory [22]. Given that ethanolic extractions of propolis are essentially chemically enriched fractions because they contain no wax, it is unclear when using raw propolis how much of the material is bioactive. Furthermore, the propolis used in the study was a year old and so likely lost a portion of its bioactive compounds. With all of this in mind, effects of more natural applications of propolis versus *Varroa* need to be explored. Furthermore, differences in the acaricidal effects of German and Argentinian propolis in these studies may be due to the differences in the concentration of compounds, the botanical origin and, more importantly, the chemical properties of the propolis from these two regions. With respect to colony-level differences in chemical composition of propolis, Popova et al. [106] showed that propolis from colonies able to maintain very low mite infestation levels without acaracide treatment were distinct in its chemical composition compared to propolis from colonies with high level of mites. Propolis from mite-tolerant colonies had significantly higher concentration of four compounds (i.e., caffeic acids and pentenyl caffeates). Borba et al. [20] found no differences in mite levels between large field colonies with a propolis envelope and those without an envelope over two years of study.

### 5.3 Viruses

Related to *Varroa* are the numerous honey bee viruses that can be transmitted through mite infestation, but also are transmitted horizontally from worker to worker or vertically via the queen. Since propolis has known activity against several viruses of human interest (reviewed in [62]), there has been considerable interest in potential effects of propolis against bee viruses, despite little study on the subject. Current evidence suggests that the presence of a propolis envelope within the honey bee nest cavity may not influence viral loads. Borba et al. [20] found no difference in the viral loads of deformed wing virus (DWV), black queen cell virus or Israeli acute bee paralysis virus in bees from propolis-rich or propolis-poor colonies in fall or spring over two years of study. However, a recent study suggests that increased propolis in colonies may impact the levels of DWV, but not sacbrood virus [22]. When raw propolis was added to the tops of colonies, DWV levels did not increase with mite infestation as was expected and observed in the propolis-deprived colonies [22]. Furthermore, they show data that indicates that colonies increase the rate of resin foraging in relation to DWV infection. Given the low mite levels in these colonies overall and the fact that they were previously treated with miticides, these intriguing findings warrant further investigation. Particularly, future work should aim to disentangle the combined effects of DWV-infection and *Varroa* infestation on resin foraging behaviour.

### 5.4 Other Hive Pests

In regard to other hive pests, two studies have examined the effectiveness of propolis extracts against the greater wax moth (*Galleria mellonella*), an opportunistic pest that mainly affects weakened hives [107,108]. Propolis extract concentrations of 4%–10% (g propolis/100 mL ethanol) showed high toxicity to *G. mellonella* when early stage larval instars had direct contact with the propolis solution for 30 s [108]. Lower concentrations of propolis extract showed sub-lethal effects by reducing pupal metamorphosis duration. However, when fractionated propolis (80% methanol extraction of propolis followed by physical and chemical fractionation) was added to artificial diet fed to *G. mellonella*, it did not influence larval growth, and no toxic effect was observed [107]. The lack of propolis fractions toxicity to *G. mellonella* when mixed into food indicates that this species may tolerate low concentrations of phenolics in their diet. This is not unusual in lepidopterans as other species in this family have also shown tolerance to some flavonoids (quercetin and rutin [109]) and, in some cases, the presence of flavonoids in the diet improves the performance of silkworms (*Bombyx mori* [110]).

Cape honey bees, *A.m. capensis*, have been observed encapsulating the parasitic small hive beetle, *Aethina tumida*, in “propolis prisons”, which serves to prevent the beetles from successfully reproducing [111,112]. The extent of this behavior in other subspecies has not been fully investigated, but it is not a trait unique to the Cape honey bee and has been noted to be variably expressed in European honey bee colonies [113,114]. The European honey bee, *A. mellifera*, will also embalm other intruders that are presumably too large to remove from the nest after being killed [5]. Hoyt [115] observed a mouse encased in propolis and suggested that the bees covered it in propolis to prevent odor and decay from affecting the rest of the hive.

## 6. Propolis and Pesticides

Another area of special interest to the beekeeping community is the presence of contaminants in-hive products, like propolis (i.e., [116]). While commercial hives are often given a variety of chemical treatments to control various hive diseases and parasites, investigations into the residues that these may leave behind are relatively new. There is limited evidence that acaricides can occasionally be found in propolis collected from a hive [22,117,118], as well as the antibiotic (tylosin) used to treat the bacterial diseases American foulbrood and European foulbrood (two of 30 samples from China had detectable amounts [119]). Similarly low levels of pesticide residues likely from treatments on the plant sources or contaminated pollen have been detected in some propolis samples [120,121], but not in others [122,123]. Further study on the frequency and abundance of these chemicals in propolis samples needs to be conducted as well as the possible antagonistic effect that these compounds could have on the chemical constituents of propolis or possibly the synergistic effects that the residues have with those chemicals found in wax, honey and pollen stores (i.e., [124,125]).

**Figure 5:**
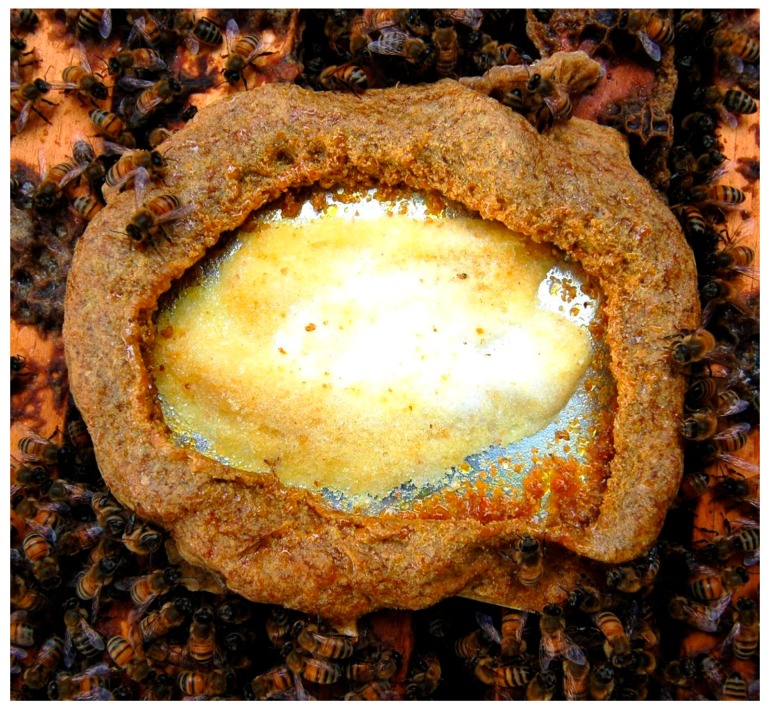
Thymol treatment on the top of a hive encased by propolis. Bees can be seen surrounding the tray with the miticide with a wall of propolis. (Photo by Michael Simone-Finstrom).

Recent evidence also indicates that honey bees may “entomb” fungicide-contaminated pollen in cells with propolis, but the frequency of this behavior and subsequent effect on colony health is currently unclear [126]. Older comb tends to have more “entombed” cells and a regular rotation of new comb into beekeeping operations would reduce the presence of these cells in colonies. It also raises the question of how bees manipulate contaminated propolis samples and they simply layer more propolis over contaminated areas. One other example seen during mite treatments with thymol products is that colonies often surround the thymol tray in a thick layer of propolis, entirely encasing it on occasion (Figure 5). It is unclear if they reuse and redistribute this propolis once the treatment is gone and if it could extend the length of the treatment or cause other negative effects at the colony level. One recent study suggests that propolis could be a “reservoir” of miticides. *Varroa* exposed in a laboratory setting to propolis harvested from a thymol-treated colony had higher mortality than mites exposed to propolis from an untreated colony, even though the propolis was tested a year after collection [22]. This result and the potential impacts warrant further study.

## 7. Propolis and Other Aspects of Bee Health

It is possible that the antimicrobial properties of materials used and stored in combs (e.g., royal jelly, honey) are enhanced by the addition of propolis [8,98]. Current work showing the effect of a propolis-enriched environment on the antimicrobial activity of larval food continues to raise this possibility [19]. Additionally, it has been suggested that some of the phenolic compounds present in honey may be derived from propolis itself [55]. Depending on how much propolis is incorporated into the comb or along the rims of cells, which is seen in feral colonies and in some managed colonies [5,127], the potential of propolis-related compounds and volatiles “leeching” into other hive products remains a possibility and needs to be further examined. Alternatively, honey bees could potentially actively “enhance” the antimicrobial activity of propolis or these other hive products by combining bee-produced compounds with the plant-produced resins, as has recently been documented in wood ants [128]. This potentially adds another dynamic to the fact that glucose oxidase (a compound produced in the mandibular glands) has been found in propolis [7]. The assumption was that it was found in propolis simply as an artefact of handling, but perhaps this needs to be investigated more fully.

In particular, a series of studies has documented the role of one particular compound, *p*-coumaric acid [56]. *P*-coumaric acid is found in pollen and nectar but is also a common component of propolis worldwide [60,75]. The ingestion of *p*-coumaric acid by honey bees increases expression of detoxification genes and increases metabolism of common beekeeper-applied miticides [56]. In addition, bees fed a diet of *p*-coumaric and another phytochemical, quercetin, also exhibited increased lifespans in some cage scenarios and after exposure to pyrethroid insecticides, though effects were complex based on the addition of protein supplements [53]. It may be that the presence of propolis in the hive and compounds like *p*-coumaric acid actually primes the detoxification pathways so that, upon exposure to certain pesticides, the honey bee is more quickly and effectively able to induce a detoxification response [55]. Ingestion of propolis extracts also decreases mortality of bees exposed to aflatoxins produced by fungi like *Aspergillus* that naturally grow on stored pollen in combs within colonies [29]. These toxic compounds are detoxified by cytochrome P450 activation, which is induced after ingestion of propolis and propolis-related compounds. The big question remains of whether or not simply exposure to propolis in the nest environment has these effects, since honey bees are not thought to typically ingest it.

Other than the potential of propolis as a detoxifying agent or primer of detoxification pathways, another hypothesized mechanism that could influence honey bee longevity via antioxidant-related pathways regards increased resistance to oxidative stress. The free radical theory of aging [129,130] bases the aging process on the production of reactive oxygen species (ROS) via typical cellular metabolism. These ROS result in the oxidation of lipids, proteins and even DNA, disrupting cellular membrane stability and eventually causing apoptosis. Aging, in some ways, is thought to be an accumulation of all of this damage due to oxidative stress. Propolis extracts, through various studies in mainly vertebrate models [131,132], have been shown to widely inhibit the formation of reactive oxygen species, which reduces oxidative damage to proteins, lipids and DNA (reviewed in [133]). Reducing damage from oxidative stress accumulation through diet, by consuming antioxidant rich foods or supplements has been sought as a way to reduce these effects of aging with limited success in humans [130]. However, antioxidants need to get into cells to quench ROS, so studies examining appropriate delivery mechanisms are important. Propolis, as a natural mixture rich in antioxidants, is certainly understudied in regard to its potential effects against oxidative stress. Impacts of oxidative stress are natural in the progression of aging even in honey bees [134,135,136] and are known to be influenced by the environmental conditions in which colonies are maintained [137]. Work on potential effects that propolis may have on colonies in a commercial beekeeping operation to help reduce possible impacts of some of the stressors, including oxidative stress, are important moving forward.

## 8. Applications for the Beekeeping Community

Several studies have now clearly documented the benefits of a propolis envelope, particularly an envelope naturally constructed by the bees, to bee health and immune system functioning. However, the effects of propolis enrichment in colonies managed in a commercial setting need to be explored more fully. The collection of resins to construct a natural propolis envelope is performed by a rare subset of foraging bees, so increased resin use should not negatively affect honey production, and, in fact, the opposite effect has actually been observed [47,48]. It is estimated that the number of resin foragers is less than 1% of the total number of foragers in the hive, but this may be influenced by the bees’ genetics [138,139]. Resin collection is partly a genetic tendency and partly a demand-driven process [16,140,141]. How and what resin collectors detect inside the nest to determine the need for resin is not clear. When resin foragers encounter rough surfaces and gaps inside the hive, they respond by collecting more resin to seal these cracks in the nest architecture and resin foragers are more sensitive to tactile information [142]. Therefore, a colony of bees can be encouraged to build a natural propolis envelope within standard beekeeping equipment by modifying the inner walls of bee boxes. Commercial propolis traps can be cut to fit the four inside walls of the hive boxes and stapled with the smooth side of the trap facing the wood and the rough side facing the colony ([20]; Figure 2B). It is recommended to manage colonies using nine frames instead of ten when using this method due to the space required for the traps. If the inside of the bee box is built with unfinished, rough lumber, scraped briskly with a wire brush, or if small grooves are cut in the interior walls of the box, the bees will apply a layer of propolis in the grooves of rough surfaces, forming a natural propolis envelope.

With this in mind, however, we must consider how altering the nest structure can also potentially adversely affect colony dynamics. The initial experimental design for the studies on the long-term effects of the propolis envelope consisted of three treatments: colonies with no extra propolis (control), colonies with a propolis envelope (Figure 2B), and colonies fitted with a propolis trap on top of the frames of the top box, as is done to collect propolis commercially. Bees from colonies with the propolis traps on top of the frames showed inconsistent, and sometimes higher immune-related gene expression, compared to bees in the propolis envelope and control colonies [19]. Moreover, bees from colonies with a propolis trap on top of the frames had significantly higher levels of virus (DWV) compared to bees in control and propolis envelope colonies in September 2012, May 2013 and May 2014 (but, see [22], which shows reduction in DWV relative to the controls). The hypothesis is that the presence of the water-resistant propolis trap throughout the year on top of the colony could have altered the microenvironment of the colony (e.g., increasing humidity levels or affecting air circulation within the nest), leading to favorable conditions for the growth of pathogens and maybe viruses. Thus, it appears that leaving a propolis trap on top of a colony for a long period of time, and, especially over the winter, is not beneficial to bee health and is not recommended. Finally, there is no evidence that bees consume resins or propolis. We do not recommend that beekeepers feed propolis solution to bees until studies adequately address the long-term effects of such a treatment. Because of the highly antibacterial and antifungal properties of propolis, it could risk poisoning bees and killing the beneficial microbiome in bees’ guts that is also so critical to their health and survival [27,82].

## 9. Conclusions

Honey bees collect plant-produced antimicrobial compounds and incorporate them into the nest environment. Honey bees, other social and solitary insects, and even some vertebrates co-opt these defensive compounds as their own form of defense. The specific role that this behavior plays in behavioral immunity is ripe for study. Here, we have described recent work that has been done on the interactions between propolis, the honey bee immune system, and honey bee pathogens, parasites and pests. Managed honey bee colonies are currently experiencing high rates of annual mortality, largely due to pathogens, parasites, pesticides and poor nutrition. Perhaps propolis can, at least in part, help mitigate effects from these threats. Understanding the role that propolis plays as a social immune defense directly against parasites and pathogens and through subtle, indirect effects on individual immunity and detoxification enzymes could be a key part of the puzzle to improve bee health. More research needs to be conducted on the long-term effects on the role of propolis on colony health and productivity in order to garner support from the beekeeping community to start selecting for propolis collection in the U.S., something that has been historically, and likely passively, selected against because of its sticky nature. In this way, propolis can be one part of an effective strategy to improve selection in U.S. stocks for resistance traits.
